# Cytokine Production and NET Formation by Monosodium Urate-Activated Human Neutrophils Involves Early and Late Events, and Requires Upstream TAK1 and Syk

**DOI:** 10.3389/fimmu.2019.02996

**Published:** 2020-01-15

**Authors:** Olga Tatsiy, Thomas Z. Mayer, Vanessa de Carvalho Oliveira, Stéphanie Sylvain-Prévost, Marilyn Isabel, Claire M. Dubois, Patrick P. McDonald

**Affiliations:** ^1^Pulmonary Division, Faculty of Medicine, Université de Sherbrooke and Centre de recherche du CHUS (CRCHUS), Sherbrooke, QC, Canada; ^2^Department of Immunology and Cell Biology, Faculty of Medicine, Université de Sherbrooke and CRCHUS, Sherbrooke, QC, Canada

**Keywords:** neutrophils, signaling, cytokines, NETs, transcription factors

## Abstract

Gout is a prevalent and incapacitating disease triggered by the deposition of monosodium urate (MSU) crystals in joints, which are also massively infiltrated by neutrophils. The interaction of the latter with MSU crystals triggers several responses, including the generation of inflammatory mediators and of neutrophil extracellular traps (NETs). Though some of the signaling events mobilized by MSU in neutrophils have been described (e.g., Src family kinases, Syk, PKC, PI3K), the picture remains fragmentary. Likewise, the impact of these signaling events on cellular responses is incompletely understood. In this study, we examined transcriptomic changes triggered by MSU in neutrophils and their impact on the corresponding proteins, as well as the role of various signaling pathways in prominent functional responses. We report for the first time that neutrophils can secrete the monocyte chemoattractant, CCL4, in response to MSU. Accordingly, we found that transcription factors NF-κB, CREB, and C/EBP are belatedly activated by MSU crystals, and at least the former is involved in chemokine generation. Moreover, we show that MAPKs and Akt are activated by MSU in neutrophils, that they are under the control of TAK1 and Syk, and that they participate in cytokine generation and NETosis. In the latter instance, we found the phenomenon to be independent of endogenous ROS, but under the control of PAD4. We finally provide evidence that endogenous factors contribute to the belated phosphorylation of kinases and transcription factors in response to MSU. Collectively, our findings unveil potentially important therapeutic targets for gouty arthritis.

## Introduction

Gout is a prevalent disease (about 1 in 50 people will develop it over a lifetime) that is very painful and incapacitating (recurring gout attacks can cause permanent joint damage). One clear distinction between gout and other arthritides is that its causative agent is known. Deposition of insoluble monosodium urate (MSU) crystals in the joint triggers an acute inflammatory reaction that is partially initiated and driven by neutrophils. Accordingly, the main mediators detected in the synovial fluid of gouty joints (i.e., IL-1β, IL-6, CXCL8, CCL3, TNFα), whether in humans (1) or in animal models (2), can all be secreted by neutrophils. More compellingly, neutrophil depletion suppresses the inflammatory response to MSU in canine joints (3, 4). Likewise, colchicine, an effective (but poorly tolerated) treatment for acute gout, potently inhibits numerous neutrophil functions (5). Together, these observations leave little doubt that neutrophils and their products represent important elements in the pathogenesis of gout.

Interactions between neutrophils and MSU crystals are known to elicit several responses. One of the first to be documented was the production of reactive oxygen species (ROS) and the concurrent release of anti-microbial peptides and proteolytic enzymes (6, 7). Neutrophils were also shown to synthesize and release the potent neutrophil chemoattractant, leukotriene B4, as well as other neutrophil chemotactic factors in response to MSU (8–11). Likewise, MSU-activated neutrophils can secrete cytokines and chemokines in response to MSU, namely IL-1β, IL-1ra, and CXCL8 (12–14). Neutrophils stimulated with MSU crystals also display a significantly delayed apoptosis (15, 16), which presumably contributes to their increased recruitment and persistence during active gouty inflammation. Finally, the ability of MSU to elicit the generation of neutrophil extracellular traps (NETs) was recently reported (17, 18).

Because of the numerous actions of MSU crystals toward neutrophils, several studies have focused on the underlying mechanisms; despite this however, our knowledge of the signaling pathways being mobilized remains fragmentary. It has been shown, for instance, that MSU rapidly triggers the phosphorylation of several neutrophil proteins on tyrosine residues, and that accordingly, tyrosine kinases such as Syk and members of the Src family are rapidly activated by the crystals in these cells (19, 20). Other kinases, namely conventional PKCs, were reported to be activated by MSU in neutrophils, and there is evidence that these PKCs can associate with Syk, resulting in its phosphorylation and interaction with PI3Ks (21, 22). Finally, studies involving pharmacological inhibitors have indicated that Src family kinases, Syk, and PI3Ks act as key signaling molecules for MSU-elicited degranulation, ROS production, generation of chemotactic activity, and NETosis in neutrophils (10, 17, 20, 21).

In view of the prevalence of gouty arthritis and of the neutrophil involvement in its pathogenesis, a better understanding of both MSU-elicited responses and of their molecular bases is clearly needed. In this regard, our previous work has provided several potential clues, insofar as we have shown the crucial involvement of TAK1, MAPKs, PI3K, and Syk in cytokine generation, delayed apoptosis, and NETosis in response to several physiological neutrophil stimuli (23–27). Under the same stimulatory conditions, we have also established that several transcription factors (e.g., NF-κB, C/EBP, CREB) drive cytokine production in neutrophils (23, 26, 28, 29). These observations raise the possibility, that some of the same kinases (in addition to Syk and PI3K) and transcription factors similarly control MSU-elicited responses. In this study, we examined the genomic and proteomic changes triggered by MSU in neutrophils, as well as the role of various signaling pathways in this and other functional responses. We now report for the first time that neutrophils can secrete CCL4 in response to MSU. Accordingly, we found that transcription factors NF-κB, CREB, and C/EBP are belatedly activated by MSU crystals, and at least the former is involved in cytokine generation. Moreover, we show that MAPKs are activated by MSU in neutrophils, that they are under the control of TAK1 and/or Syk, and that they participate in cytokine generation and NETosis.

## Materials and Methods

### Antibodies and Reagents

Antibodies against P-Akt (#4060), P-ERK (#9101), P-p38 (#9212), P-Src (#2101), P-Syk (#2711), P-C/EBPβ (#3084), P-CREB (#9191), P-RelA (#3031), IκBζ (#9244), and MAP3K8 (#4491) were all from NEB-Cell Signaling (Danvers, MA, USA); antibodies against IκB-α (sc-371) and β-actin (sc-1616) were from Santa Cruz Biotechnology (Santa Cruz, CA, USA). Ficoll-Paque Plus was from GE Biosciences (Baie d'Urfé, Qc, Canada); endotoxin-free (< 2 pg/ml) RPMI 1640 was from Wisent (St-Bruno, Qc, Canada). MSU was from Cayman Chemical (Ann Arbor, MI, USA); recombinant human cytokines were from R&D Systems (Minneapolis, MN, USA); UltraPure LPS (from *E. coli* 0111:B4) was from InvivoGen (San Diego, CA, USA). Actinomycin D, cycloheximide, culture-grade dimethyl sulfoxide (DMSO), N-formyl-methionyl-phenylalanine (fMLP), and phenylmethanesulphonyl fluoride (PMSF) were from Sigma-Aldrich (St. Louis, MO, USA). Diisopropyl fluorophosphate (DFP) was from Bioshop Inc. (Burlington, Ont., Canada). The protease inhibitors, aprotinin, 4-(2-aminomethyl)benzenesulfonyl fluoride (AEBSF), leupeptin, and pepstatin A, were all from Roche (Laval, Qc, Canada). Kinase inhibitors and fluorescent probes were purchased through Cedarlane Labs (Missisauga, Canada). PlaNET Blue reagent was from Sunshine Antibodies (https://sunshineantibodies.com/planet-002.html). All other reagents were of the highest available grade, and all buffers and solutions were prepared using pyrogen-free clinical grade water.

### Cell Isolation and Culture

Neutrophils were isolated from the peripheral blood of healthy donors, following a protocol that was approved by an institutional ethics committee (Comité d'éthique de la recherche du CIUSS de l'Estrie-CHUS). The entire procedure was carried out at room temperature and under endotoxin-free conditions, as described previously (30). Purified neutrophils were resuspended in RPMI 1640 supplemented with 5% autologous serum, at a final concentration of 5 × 10^6^ cells/ml (unless otherwise stated). As determined by Wright staining and FACS analysis, the final neutrophil suspensions contained fewer than 0.1% monocytes or lymphocytes; neutrophil viability exceeded 98% after up to 4 h in culture, as determined by trypan blue exclusion and by Annexin V/propidium iodide FACS analysis.

### Immunoblots

Samples were prepared, electrophoresed, transferred onto nitrocellulose, and processed for immunoblot analysis as previously described (26, 31).

### RNA Extractions, Real-Time PCR Analyses, and Gene Microarray Analyses

Procedures and primers used are exactly as described (28). When samples were prepared for gene microarray analysis, total RNA from 5 × 10^7^ neutrophils was isolated as described (28), purified using a Qiagen RNeasy MinElute cleanup kit, and processed for gene microarray analysis using the Affymetrix Human Gene 2.0 ST chip (Génome Québec, Montréal, QC, Canada).

### ELISA Analyses

Neutrophils (3 × 10^6^ cells/600 μl) were cultured in 24-well plates at 37°C under a 5% CO_2_ atmosphere, in the presence or absence of stimuli and/or inhibitors, for the indicated times. Culture supernatants, as well as the corresponding cell pellets, were carefully collected, snap-frozen in liquid nitrogen, and stored at −80°C. Samples were analyzed in ELISA using commercially available capture and detection antibody pairs (R&D Systems, BD Biosciences).

### NETosis Assays

The procedure used was exactly as described (27).

### Data Analysis

All data are represented as the mean ± SEM. Statistical differences were analyzed by Student's *t* test for paired data using Prism 7 software (GraphPad Software, San Diego, CA, USA).

## Results

### Transcriptomic Changes Elicited by MSU in Neutrophils, and Its Consequences on Cognate Proteins

**Figure 1:**
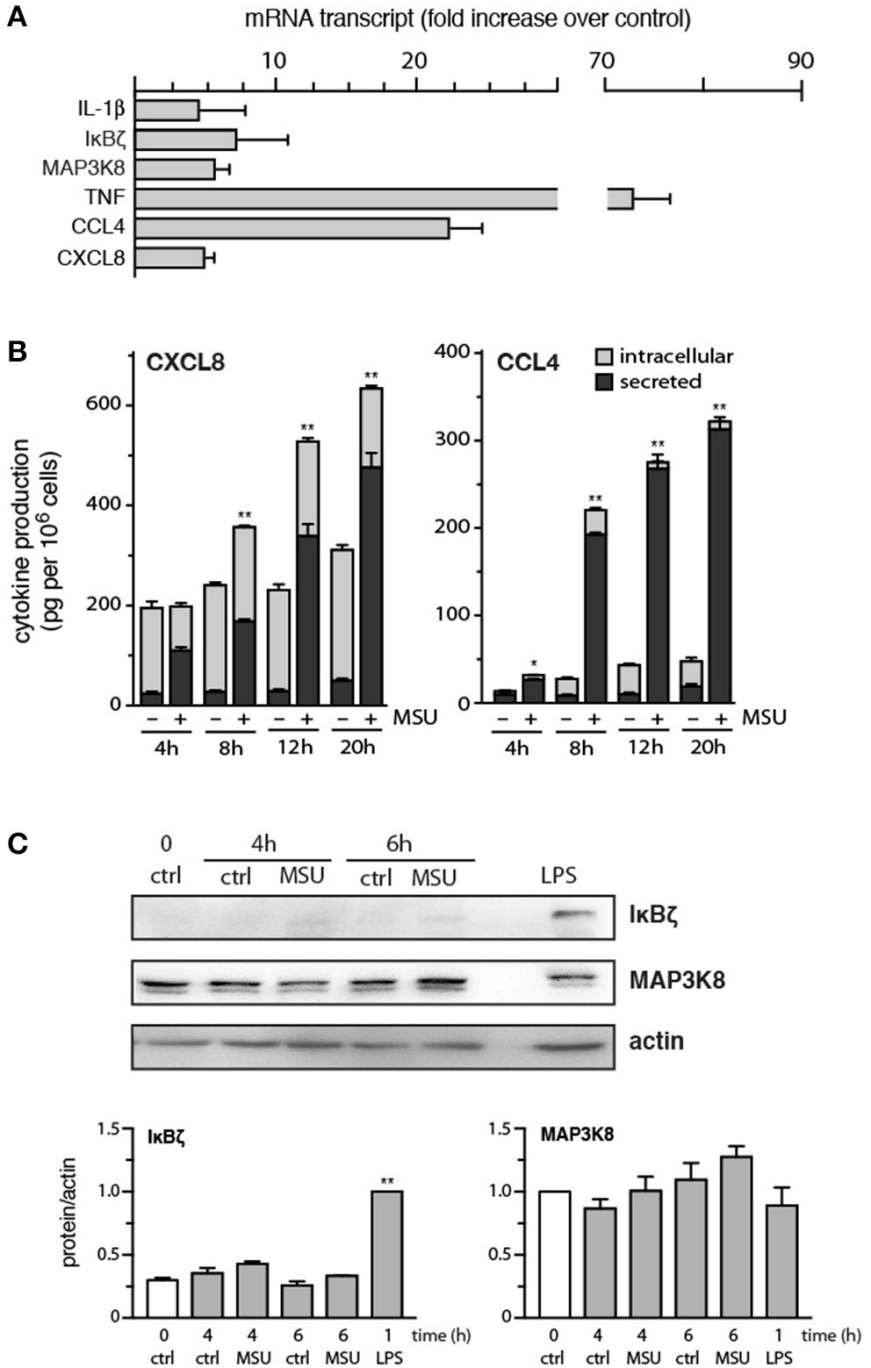
Expression of strongly induced genes, and the corresponding proteins, in MSU-activated human neutrophils. **(A)** Cells were stimulated for 3 h with 1 mg/ml MSU, prior to RNA extraction, reverse transcription, and qPCR analysis. Values were normalized over RPL32 and are represented as fold increase relative to unstimulated cells. Mean ± s.e.m. from 3 independent experiments, each performed in duplicate. **(B)** Neutrophils were stimulated with 1 mg/ml MSU for the indicated times, prior to ELISA analysis of cell-associated chemokines and of chemokines in culture supernatants. Mean ± s.e.m. from 3 independent experiments, each performed in duplicate. *p < 0.05 and **p < 0.01 for total chemokine vs. the respective unstimulated controls. **(C)** Neutrophils were cultured in the absence (“ctrl”) or presence of 1 mg/ml MSU or 1 μg/ml LPS for the indicated times, prior to immunoblot analysis of cellular IκBζ, MAP3K8, and β-actin (loading control). A representative experiment is shown, along with compiled data from at least 3 independent experiments. **p < 0.001 vs. unstimulated control.

We first revisited the issue of the genes induced by MSU crystals in neutrophils, a response that hasn't been systematically investigated to date. The cells were initially cultured for 1 h with MSU, in an effort to detect immediate-early genes, and total RNA was processed for gene microarray analysis. Disappointingly, no transcript was induced by more than 1.8 fold; likewise, no transcript was reduced by more than 2 fold (data not shown). Thus, transcriptomic changes exerted by MSU at early stimulation times are modest at best. We repeated these experiments using neutrophils stimulated with MSU for 3 h, to determine whether gene expression changes are more pronounced at later times. As shown in Supplementary Figure 1, most genes examined exhibited changes in expression that were lesser than 3 fold. Despite this, several genes encoding inflammatory products were detected, whose expression was induced 3-fold or more (vs. unstimulated cells). These included IL-1-1α/β and CXCL8, as already reported (12–14), but also included transcripts that had not yet been observed to be induced in response to MSU, such as TNFα, CCL4, and Tpl2/MAP3K8 (Supplementary Figure 2). Other genes were similarly induced, whose products are however unknown (Supplementary Figure 2). When we validated these results by qPCR, we confirmed that the TNFα, IL-1β, CXCL8, CCL4, MAP3K8, and IκBζ genes were indeed strongly induced by MSU in human neutrophils (Figure 1A).

We next investigated whether the corresponding proteins were also upregulated in MSU-treated neutrophils. Cells were cultured for increasing lengths of time with the crystals, prior to ELISA or immunoblot analysis of the proteins of interest. As shown in Figure 1B, substantial amounts of CXCL8 and CCL4 were synthesized and secreted over time. Initially, most of the released CXCL8 came from preformed pools of the chemokine, whereas the later secretion of CXCL8 predominantly involved newly synthesized CXCL8 (Figure 1B). This is in contrast with the secretion of CCL4, which largely reflects the accumulation of newly-made chemokine (Figure 1B). By comparison, IL-1α/β or TNFα production was either undetected or at the detection limit at 20 h (data not shown). Finally, cellular levels of MAP3K8 were not significantly affected in MSU- or LPS-activated cells (Figure 1C). Cellular expression of IκBζ was also unchanged following MSU stimulation, though LPS did induce an accumulation of the protein, as expected (Figure 1C).

### Signaling Cascades That Are Rapidly Elicited by MSU

**Figure 2:**
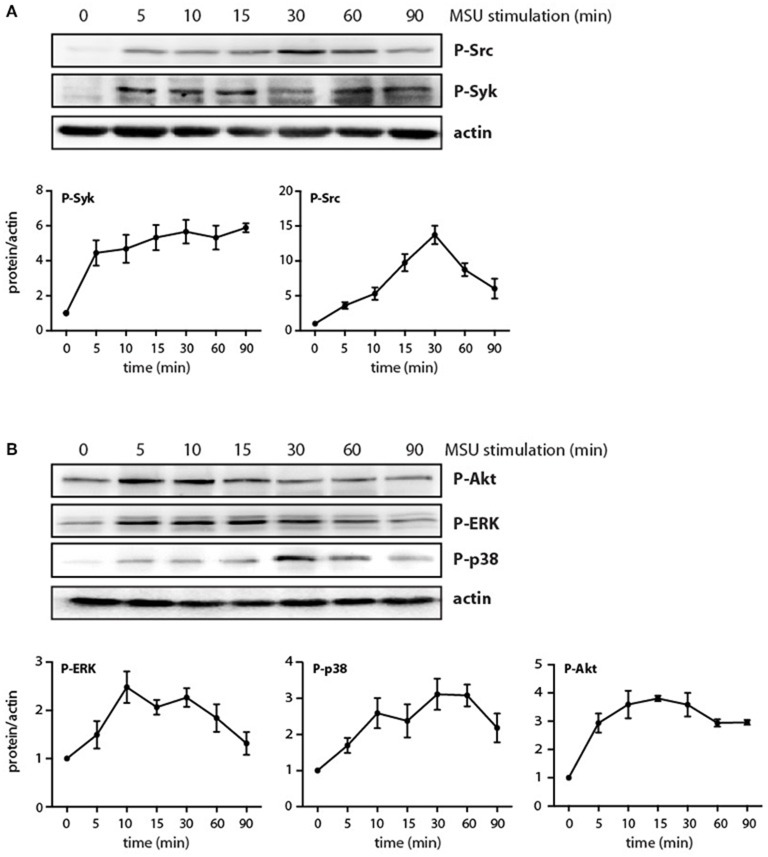
Phosphorylation of signaling intermediates in MSU-stimulated neutrophils. Cells were stimulated with 1 mg/ml MSU for the indicated times, prior to immunoblot analysis of **(A)** cellular P-Src^Y416^ or P-Syk^Y525/526^; **(B)** P-Akt^S473^, P-ERK, or P-p38 MAPK; and β-actin (as a loading control). A representative experiment is shown in both panels, along with compiled data from at least 3 independent experiments.

Although some signaling intermediates are known to be activated by MSU in neutrophils, the picture remains incomplete; likewise, their eventual role in neutrophil functional responses needs be elucidated. When we monitored the kinetics of various signaling pathways in MSU-treated neutrophils, we confirmed that the Src and Syk pathways are quickly activated in these cells, with phosphorylated kinases slowly returning to near-baseline levels by 90 min in the case of Src, but still elevated in the case of Syk (Figure 2A). We additionally found that MSU-stimulated neutrophils display a rapid activation of the PI3K/Akt, p38 MAPK, and ERK pathways (Figure 2B), with Akt showing sustained phosphorylation at 90 min, whereas p38 MAPK and P-ERK activation appeared to be more transient. By contrast, no changes were observed in cellular IκB-α levels; similarly, phosphorylated JNK was undetected; and little or no inducible phosphorylation of the transcriptional activators, RelA, C/EBPβ, and CREB, were observed under these conditions (data not shown). Thus, a discrete set of signaling pathways seem to be mobilized by MSU in neutrophils.

**Figure 3:**
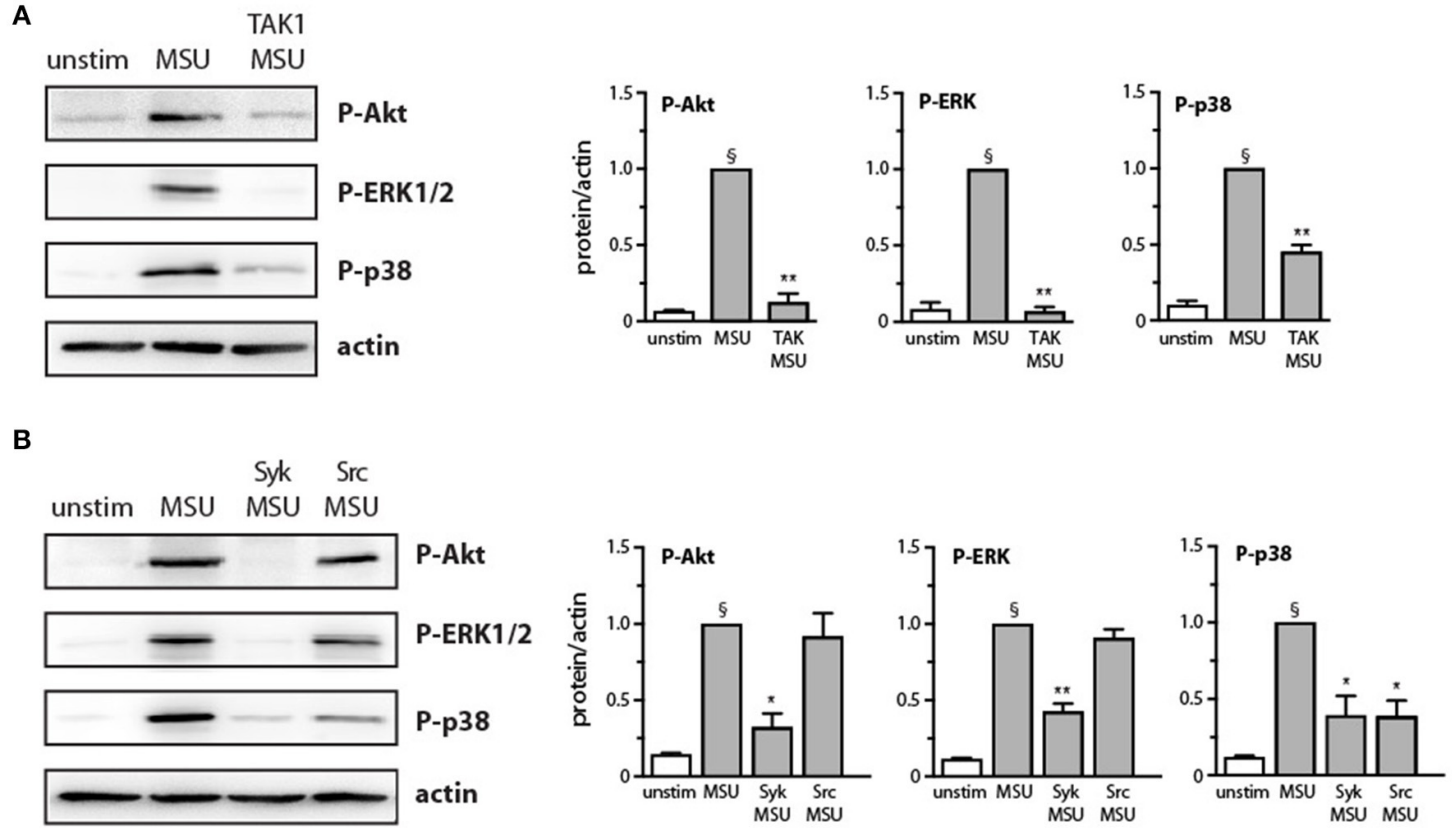
Effect of Src, Syk, and TAK1 inhibition on Akt and MAP kinase activation in MSU-stimulated human neutrophils. Cells were pretreated for 10 min in the absence or presence of **(A)** a TAK1 inhibitor [1 μM 5(Z)-7-oxozeaenol] or **(B)** a Src inhibitor (10 μM SrcI1) or a Syk inhibitor (10 μM piceatannol), prior to stimulation for 15 min with 1 mg/ml MSU or diluent control (“unstim”). Whole-cell samples were processed for immunoblot analysis of P-Akt^S473^, P-ERK, P-p38 MAPK, or β-actin (as a loading control). Representative experiments are shown, along with compiled data from at least 3 independent experiments. ^§^*p* < 0.003 vs. unstimulated controls; *p < 0.05 and **p < 0.01 vs. MSU alone.

We have shown previously that the p38 MAPK, MEK/ERK, and PI3K/Akt cascades are controlled by the MAP3K, TAK1, in human neutrophils exposed to various physiological stimuli (24, 25, 32, 33). We therefore verified whether this is also the case in response to MSU crystals. As shown in Figure 3A, TAK1 inhibition mostly blocked the phosphorylation of all three kinases in response to MSU. We also reported that Syk and Src family tyrosine kinases can affect at least some neutrophil responses (26, 27) and our observation that MSU rapidly activates these kinases (Figure 2A) prompted us to examine whether they may also act upstream of MAPKs and Akt. As shown in Figure 3B, Syk inhibition profoundly hindered the phosphorylation of all three kinases, while Src inhibition only significantly affected that of p38 MAPK. Thus, both TAK1 and Syk act upstream of MAPKs and Akt, while Src family kinases contribute only to p38 MAPK activation.

### Impact of Signaling Cascades on MSU-Elicited Cytokine Production, and Occurrence of Late Signaling Events

**Figure 4:**
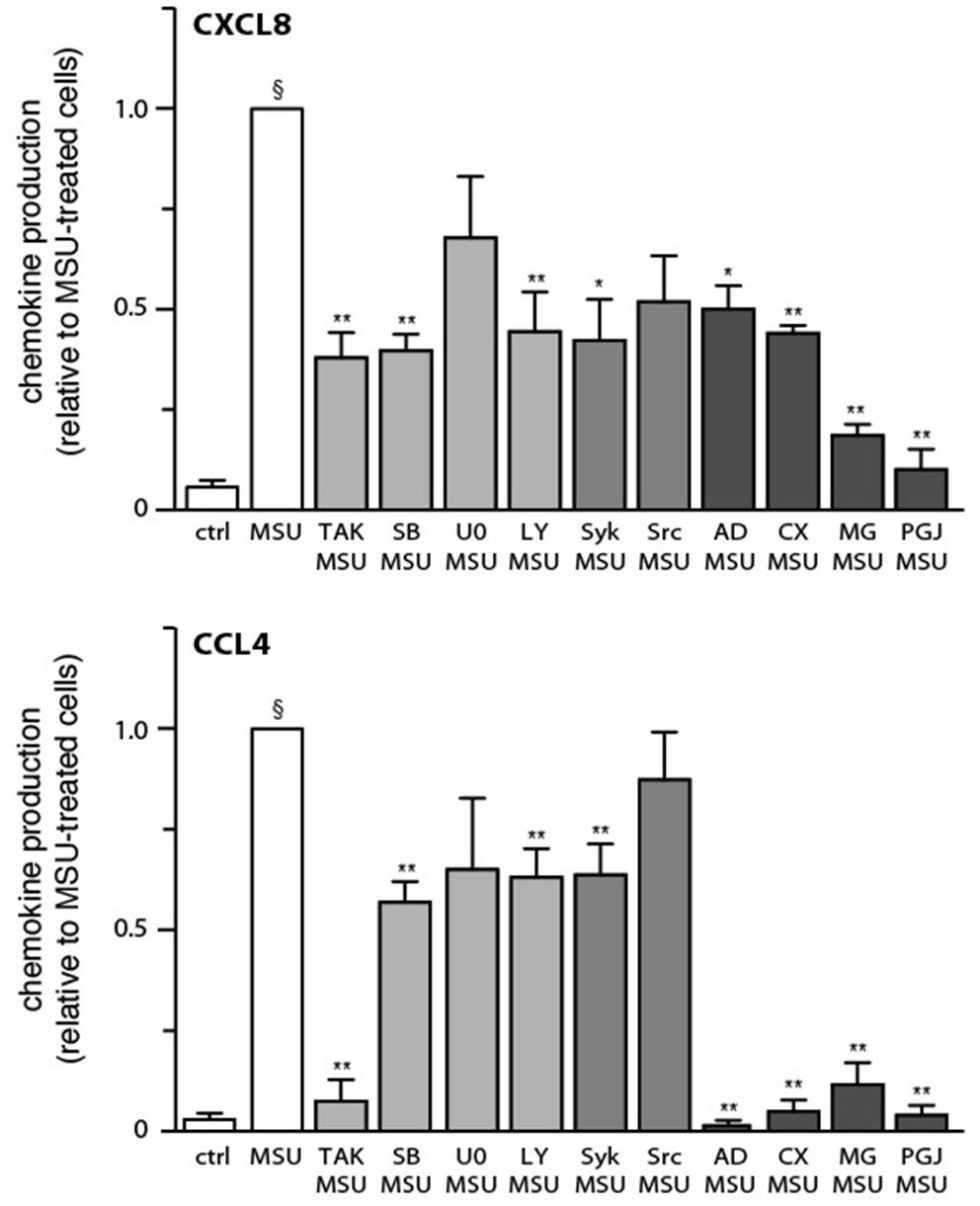
Effect of various inhibitors on chemokine secretion in MSU-stimulated human neutrophils. Cells were pretreated for 10 min in the absence or presence of inhibitors of TAK1 [1 μM 5(Z)-7-oxozeaenol], p38 MAPK (1 μM SB202190), MEK (10 μM U0126), PI3K (10 μM LY294002), Syk (10 μM piceatannol), Src family kinases (10 μM SrcI1), transcription (5 μg/ml actinomycin D, “AD”), protein synthesis (20 μg/ml cycloheximide, “CX”), or NF-κB (1 μM MG-262 or 30 μM 15-deoxy-PGJ2). Neutrophils were then cultured in the absence (“ctrl”) or presence of 1 mg/ml MSU for 20 h, prior to ELISA analysis of culture supernatants. Mean ± s.e.m. from 11 independent experiments, each performed in duplicate. Data is expressed as a ratio to MSU-stimulated cells, which amounted to 815 ± 60 pg/10^6^ cells for CXCL8, and 375 ± 63 pg/10^6^ cells for CCL4. ^§^*p* < 0.0001 vs. unstimulated controls; *p < 0.05 and **p < 0.01 vs. MSU alone.

**Figure 5:**
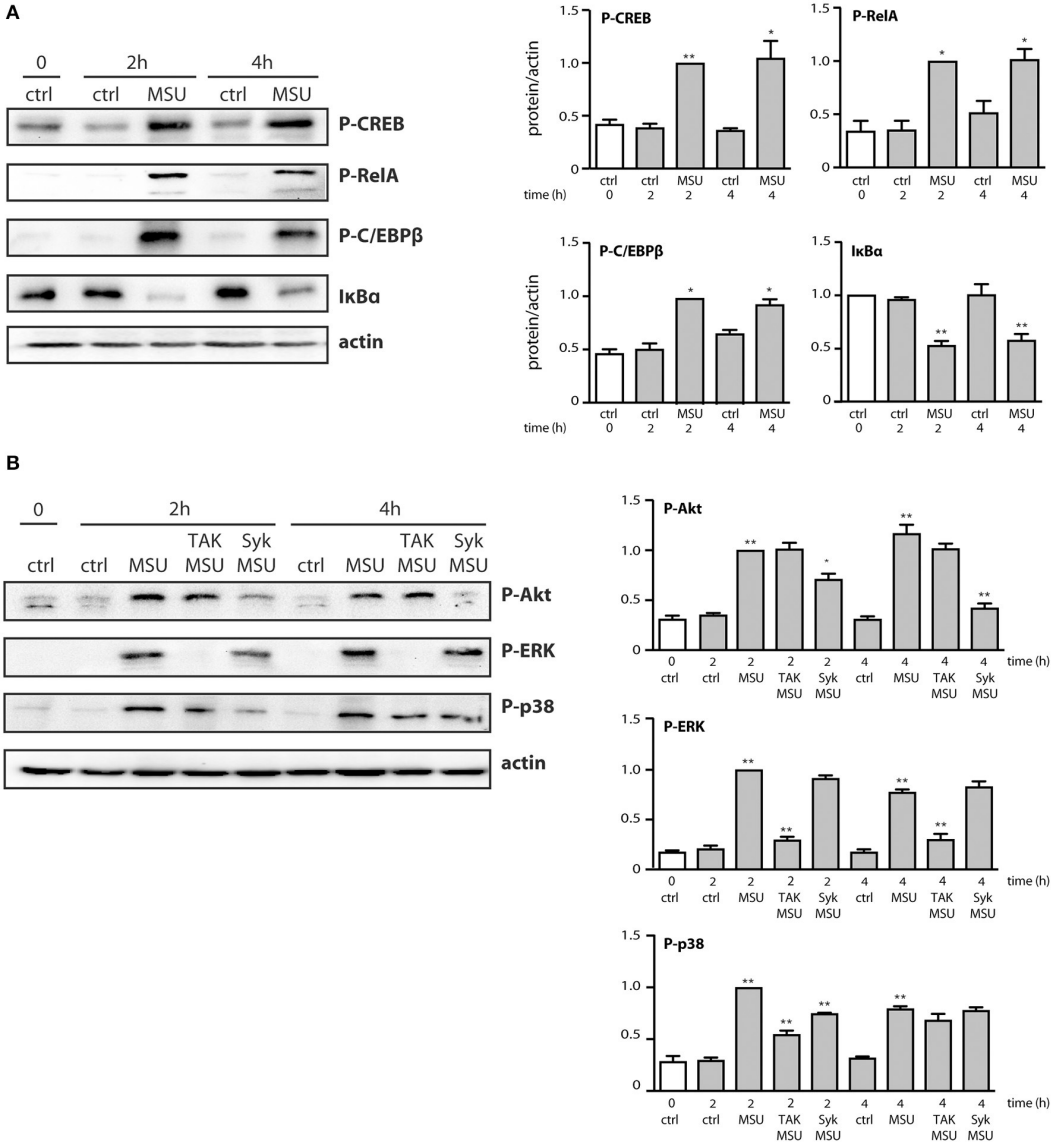
Belated phosphorylation of transcription factors and associated proteins in MSU-stimulated human neutrophils. Cells were stimulated for the indicated times in the absence (“ctrl”) or presence of 1 mg/ml MSU, prior to immunoblot analysis of cellular P-CREB^S133^, P-RelA^S536^, P-C/EBPβ^T235^, IκB-α, and β-actin (as a loading control). **(A)** A representative experiment is shown, along with compiled data from at least 3 independent experiments. *p < 0.05 and **p < 0.01 vs. respective unstimulated controls. **(B)** Neutrophils were pretreated for 10 min in the absence or presence of a TAK1 inhibitor [1 μM 5(Z)-7-oxozeaenol] or a Syk inhibitor (10 μM piceatannol), prior to stimulation for the indicated times with 1 mg/ml MSU or diluent control (“ctrl”). Samples were then processed for immunoblot analysis using P-Akt, P-ERK, P-p38 MAPK, or β-actin (as a loading control). A representative experiment is shown, along with compiled data from 3 independent experiments. *p < 0.05 and **p < 0.01 vs. respective unstimulated controls.

We next determined which signaling pathways contribute to MSU-induced cytokine production. To this end, neutrophils were pretreated with various inhibitors, prior to stimulation for 20 h. As shown in Figure 4, inhibition of TAK1, p38 MAPK, PI3K, and Syk impaired the generation of both CXCL8 and CCL4. In contrast, inhibition of the MEK/ERK or STK pathways had no significant effect on chemokine release (Figure 4). Blocking protein synthesis with cycloheximide, or transcription with actinomycin D, confirmed that MSU-elicited chemokine secretion largely depends on their *de novo* synthesis and gene expression, respectively (Figure 4). In addition, we found that pretreating neutrophils with the NF-κB blockers, MG-132 or 15-deoxy-PGJ2, profoundly ihibited chemokine production (Figure 4). This was quite unexpected, as both inhibitors target IκB-α degradation, which we had found not to occur following MSU exposure, at least over the first 60 min of stimulation (data not shown). This notwithstanding, we also observed that few mRNA transcripts accumulate in reponse to MSU in that time frame, requiring 3 h instead to be detected in abundance (Supplementary Figures 1, 2). This prompted us to investigate whether transcription factors (and associated proteins) might be activated at later time points. As shown in Figure 5A, IκB-α degradation was evident by 2 h in MSU-treated neutrophils, and IκB-α levels had still not been replenished at 4 h of stimulation. An inducible phosphorylation of transcription factors RelA, C/EBPβ, and CREB was also found to follow a similar time course (Figure 5A). Thus, a belated induction of transcriptional events takes place in MSU-activated neutrophils, in keeping with the delay in gene expression.

**Figure 6:**
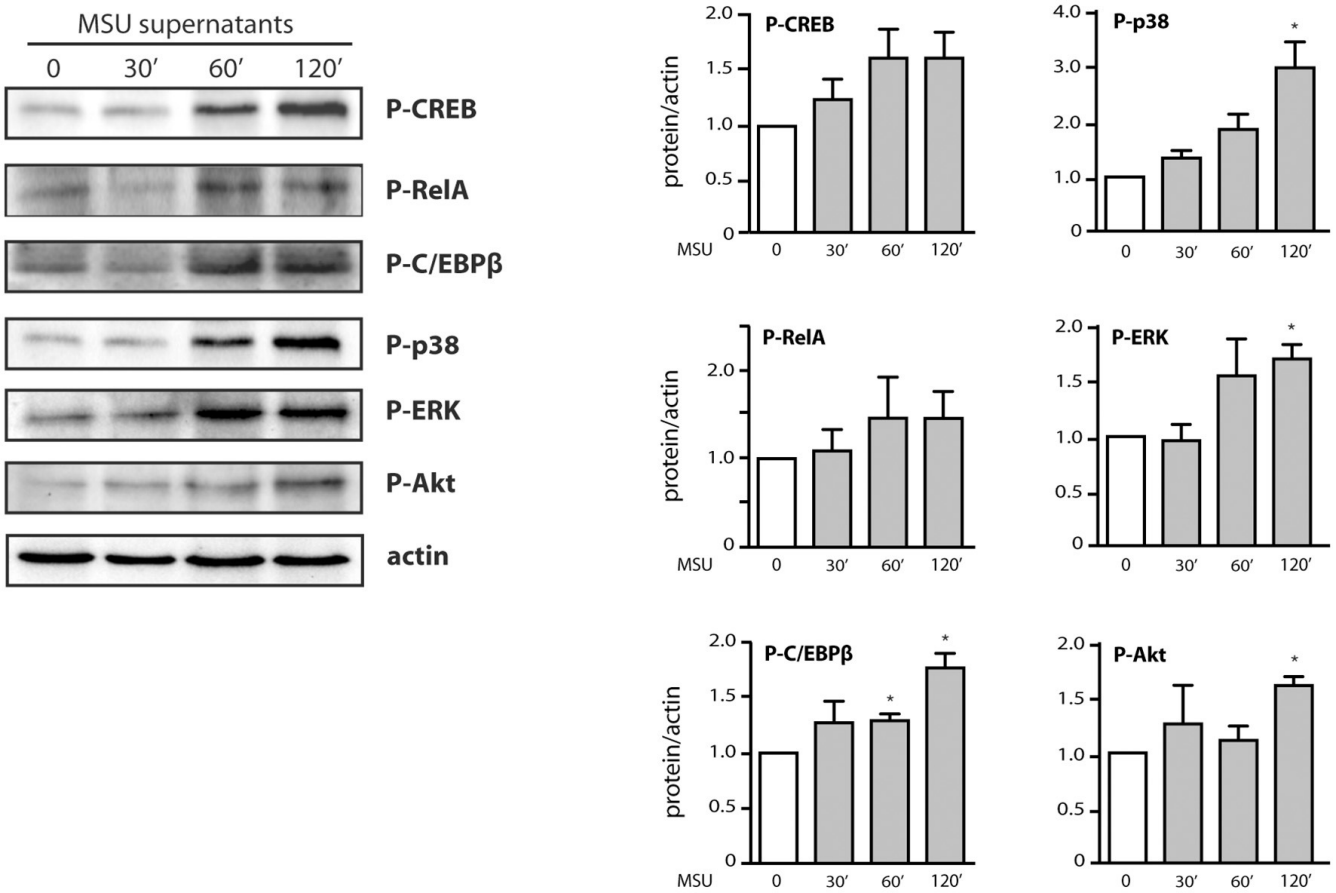
Contribution of endogenous factors to the belated transcription factor and kinase activation observed in MSU-stimulated neutrophils. Cells were incubated for the indicated times in the presence of 1 mg/ml MSU; culture supernatants were collected, depleted of MSU crystals by centrifugation (15,000 g, 10 min), and incubated with fresh neutrophils for 10 min. Samples were then processed for immunoblot analysis of cellular P-CREB^S133^, P-RelA^S536^, P-C/EBPβ^T235^, P-Akt^S473^, P-ERK, P-p38 MAPK, and β-actin (as a loading control). A representative experiment is shown, along with compiled data from at least 3 independent experiments. *p < 0.05 vs. cells incubated with control supernatants.

We also found that in parallel to transcription factor phosphorylation, some kinases involved in cytokine production (e.g., p38 MAPK, Akt) were still phosphorylated at later time points (Figure 5B). However, they were decreasingly under the control of TAK1 or Syk (Figure 5B), compared to shorter stimulation times (Figure 3). By contrast, phosphorylated ERK remained firmly under the control of TAK1 but lost its dependence on upstream Syk (Figure 5B). The occurrence of phosphorylated transcription factors and kinases at late time points prompted us to investigate whether endogenously released factors might account for the phenomenon. To this end, neutrophils were stimulated for up to 2 h with MSU and the resulting culture supernatants were collected, depleted of MSU crystals, and used to stimulate fresh neutrophils for 10 min. As shown in Figure 6, supernatants from MSU-activated cells contain endogenous material that promotes transcription factor and kinase phosphorylation; this was especially evident in supernatants from cells that were stimulated for 2 h with MSU.

### Signaling Cascades Involved in MSU-Elicited NETosis

**Figure 7:**
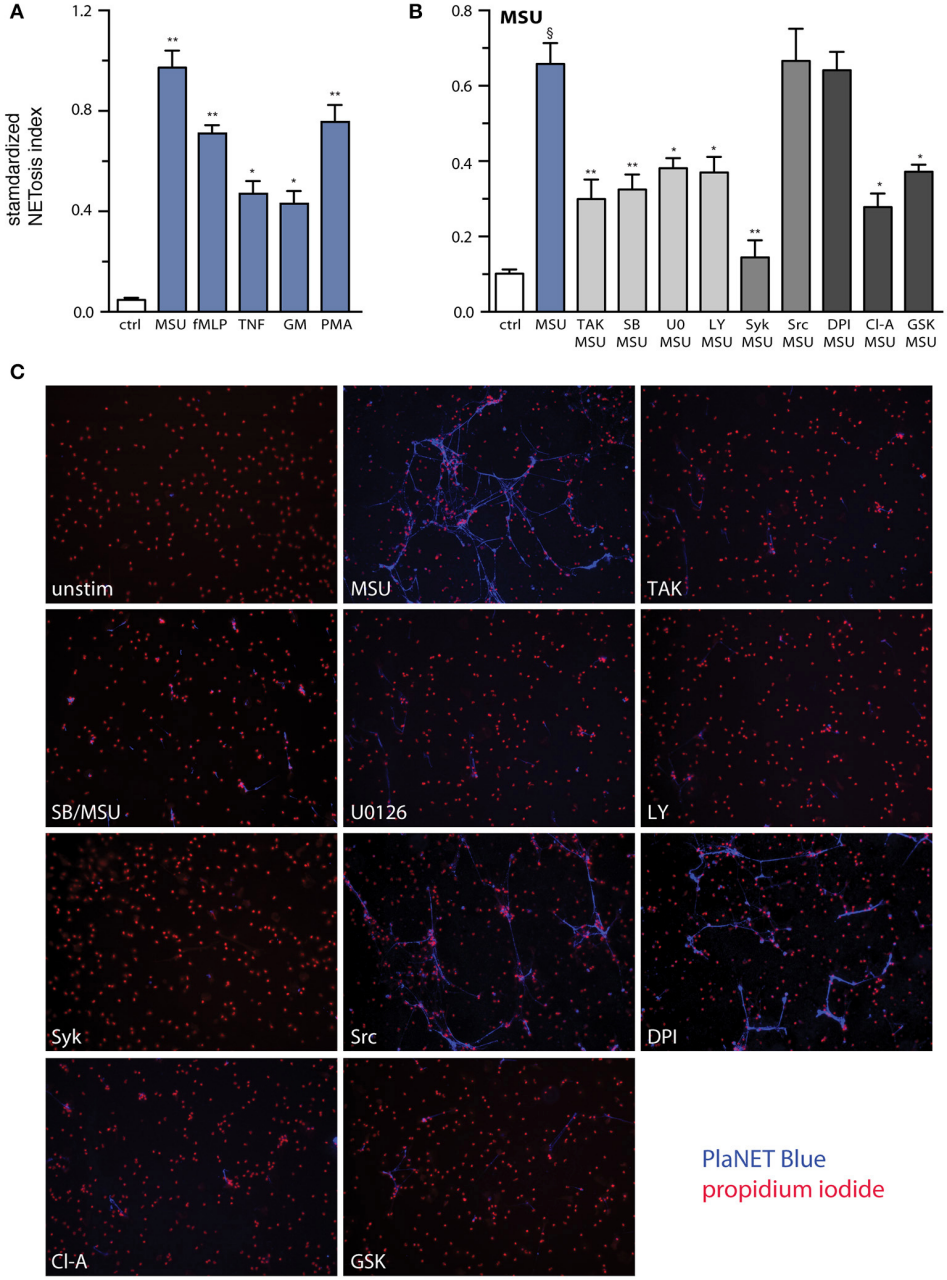
Relative potency of MSU as a NET inducer, and signaling pathways controlling this response. **(A)** Neutrophils cultured on poly-L-lysine-coated coverslips were incubated for 4 h in the absence (“ctrl”) or presence of 1 mg/ml MSU, 30 nM fMLP, 100 U/ml TNFα, 1 nM GM-CSF, or 50 nM PMA. NETosis was assessed using PlaNET Blue as described in *Methods*. Mean ± s.e.m. from 3 independent experiments. *p <0.02 and **p< 0.01, relative to unstimulated cells. **(B)** Neutrophils cultured as described in **(A)** were pre-treated (15 min, 37°C) with the following inhibitors or their diluent (culture-grade DMSO): 1 μM (5Z)-7-oxozeaenol (TAK1 inhibitor); 1 μM SB202190 (p38 MAPK inhibitor); 10 μM U0126 (MEK inhibitor); 10 μM LY294002 (PI3K inhibitor); 10 μM piceatannol (Syk inhibitor); 10 μM SrcI1 (Src family kinase inhibitor); 10 μM DPI (a NADPH oxidase inhibitor); 10 μM chloraminidine (“Cl-A,” a general PAD inhibitor); or 10 μM GSK484 (a PAD4 inhibitor). The cells were then further incubated for 4 h in the absence (“ctrl”) or presence of 1 mg/ml MSU. NETosis was assessed using PlaNET Blue as described in *Methods*. Mean ± s.e.m. from at least 4 independent experiments. ^§^*p* < 0.002 vs. unstimulated control; *p < 0.05 and **p < 0.01 vs. stimulus alone. **(C)** Representative fields for each experimental condition shown in **(B)**, at 10X magnification.

Besides cytokine production, another major functional response of neutrophils is their ability to form NETs (34). This phenomenon was reported to occur in response to MSU crystals (17, 18, 35, 36). Using PlaNET reagents, which allow a specific, standardized assessment of NETosis (27), we confirmed these findings and could further establish that MSU represents the most potent NET inducer which we ever tested, even when compared to stalwarts like fMLP or PMA (Figure 7A). To determine which MSU-elicited signaling pathways influence NETosis, neutrophils were pretreated with various inhibitors, prior to being cultured with MSU. As shown in Figure 7B, inhibition of the TAK1, p38 MAPK, MEK, PI3K, and Syk pathways partially or totally prevented NET generation, whereas blocking Src family kinases had little or no effect on this response (Figure 7B). Because NETosis was initially thought to depend on endogenous ROS, and because MSU has long been known to promote the formation such molecules in neutrophils (37), we investigated whether blocking the NADPH oxidase would interfere with NET generation. As shown in Figures 7B,C, MSU-elicited NETosis was found to be ROS-independent, but it was largely prevented by inhibition of PAD4. Collectively, the above findings shed more light on the pathways and processes controlling NETosis in MSU-stimulated neutrophils.

## Discussion

Various aspects of the interaction between MSU crystals and inflammatory cells involved in gout pathogenesis have been studied in the last decades. Despite this, many gaps in our knowledge remain. In this study, we revisited the genomic changes triggered by MSU in neutrophils, their impact on the corresponding proteins, and the signaling pathways controlling MSU-elicited functional responses. This allowed us to uncover a new chemokine secreted in response to MSU; three trancription factors belatedly activated by the crystals; and signaling intermediates acting upstream of cytokine generation and NET formation.

Though some neutrophil genes were shown to be induced by MSU over the years, a systematic investigation of transcriptomic changes was (somewhat surprisingly) never undertaken. Herein, we found that unlike most neutrophil stimuli, which induce early gene expression within 30 min, MSU does not even modulate mRNA steady-state levels 2-fold over a 60-min stimulation. After 3 h however, the expression of numerous transcripts was up- or down-regulated. Among those whose accumulation was induced 3-fold or more were previously reported transcripts such as IL-1α/β and CXCL8 (12–14), but also others that had never been observed before. Among the latter, some encode inflammatory mediators (e.g., CCL4, TNFα) or signaling machinery components (e.g., MAP3K8, IκBζ). Yet this still represents relatively few genes overall, especially when compared to classical neutrophil stimuli (such as LPS or TNFα) which, unlike MSU, strongly promote the expression of dozens of genes. Compounding the relative paucity of transcripts induced by MSU, is our observation that even fewer of the corresponding proteins actually accumulate. A striking example is that of TNFα, whose gene was induced some 70-fold, yet without any detectable accumulation of intracellular cytokine. This raises the intriguing possibility, that MSU fails to fully mobilize the translational machinery of neutrophils; studies are in progress to elucidate this conundrum. Whatever the case may be, our data represents the first report that CCL4 can be secreted by MSU-treated neutrophils. This finding has potentially important biological implications, insofar as MSU-activated neutrophils can not only contribute to their own recruitment into inflamed joints by generating CXCL8, but can also attract monocytes through their ability to secrete CCL4. In keeping with this notion, both neutrophils and monocytes are recruited by MSU crystals.

The signaling events triggered by MSU crystals in neutrophils have been only partially elucidated to date. It has been shown, for instance, that Src family kinases, Syk, PKCs, and PI3Ks are activated upon MSU challenge (19–22). We confirmed herein that Syk and Src are rapidly phosphorylated in response to MSU; whereas this response was sustained for of P-Syk (for at least 90 min), it was transient in the case of P-Src. Importantly, we found that p38 MAPK, ERK, and Akt were also rapidly phosphorylated in MSU-stimulated cells, and that the phospho-proteins were still detected after 90 min. In the case of p38 MAPK, our data confirm and extend recent observations by Rousseau et al. (38), who however only detected weak p38 phosphorylation over a 5-min interval. By comparison, our data represents the first demonstration that ERK and Akt^Ser473^ can also be activated by MSU. Thus, the kinases activated by MSU are essentially the same as those mobilized by several physiological neutrophil agonists (23, 25, 26, 33, 39, 40). Morevoer, we found that the MSU-elicited phosphorylation of p38 MAPK, ERK, and Akt occurs downstream of TAK1 and Syk, much like it does in response to several classical neutrophil stimuli (24, 33). Thus, the undetectable synthesis of several proteins despite strongly induced corresponding genes in MSU-treated cells, cannot be attributed to a general defect in signaling. However, we observed that the extent to which Syk, Src, MAPKs, and Akt are phosphorylated is lower in response to MSU crystals, compared to classical stimuli such as LPS and TNFα. This notwithstanding, we showed that the Syk, TAK1, p38 MAPK, MEK/ERK, and PI3K/Akt pathways all contribute to chemokine generation and/or NETosis. Therefore, even a relatively weaker activation of these kinases by MSU is sufficient to entail functional consequences. On final note, it has been reported that the MSU-elicited synthesis and secretion of IL-8 in monocytes is dependent on the activity of Src kinases and of ERK1/2 (41, 42), whereas we found herein that Src inhibition had little impact on CXCL8 generation in neutrophils. This indicates that among the various signaling pathways mobilized by MSU, different combinations contribute to a given response depending on the cell type.

Another novel finding reported herein is that the NF-κB, C/EBP, and CREB transcription factors are activated in response to MSU crystals in neutrophils. This agrees well with the fact that both CXCL8 and CCL4, whose transcripts and proteins are also induced by MSU, feature cognate binding sites for these transcription factors in their proximal gene promoters, that are required for induction in human granulocytes (28, 29, 43). A singular characteristic of transcription factor activation by MSU, is that it was never detected at early time points (i.e., within 15 min), as is the case with other neutrophil stimuli, such as LPS, TNFα, or IL-18 (28, 29, 31, 39). Instead, phosphorylation of RelA, C/EBPβ, and CREB1, as well as IκBα degradation, were only observed at 120 min and beyond. This belated activation mirrors the delayed induction of chemokine genes occurring in response to MSU, which was detected at 3 h. This is again in contrast with stimuli such as LPS, TNFα, or IL-18, which typically promote chemokine gene induction within 30 min or less. Thus, whereas a similar set of transcription factors can be activated by cytokines, TLR ligands, and MSU in neutrophils, the latter stimulus does so belatedly, resulting in the late induction of target genes. This is not due to a slow ingestion of the crystals, as the process takes place within 15 min (44). On the contrary, the delayed mobilization of the transcriptional machinery, and even more so the sustained activation of MAP kinases and Akt at late time points, seem to reflect (at least in part) the production of endogenous mediators. Supportive evidence stems from our observation, that the addition of supernatants from neutrophils stimulated with MSU for 120 min consistently induced the phosphorylation of kinases (p38, ERK, Akt) and of at least some transcription factors. Studies are in progress to determine the nature of the endogenous factors involved.

Finally, MSU crystals proved to be the most powerful NET inducers that we ever tested. Whereas other investigators had already reported that this response requires the PI3K, RIPK, and MLKL pathways (17, 45), we showed herein that it also involves the TAK1, p38 MAPK, MEK/ERK, and Syk pathways. With regard to the cellular processes governing NET formation, we observed that MSU-elicited NETosis is independent of ROS generation, confirming recent reports (36, 46, 47). Conversely, our finding that MSU-induced NET formation depends on PAD4, is to our knowledge a first. Thus, MSU appears to function like most other physiological neutrophil agonists (e.g., TNFα, GM-CSF, fMLP, PAF, C5a, CXCL8) with respect to the involvement of endogenous ROS and PAD4 (27). Overall, our findings substantially extend our understanding of the mechanisms underlying NET generation, by showing that MSU crystals represent yet another class of physiological stimuli (in addition to growth factors, chemoattractants, and cytokines) (27) that employ common signaling components, as well as PAD4.

In summary, MSU crystals elicit a robust induction of a limited set of genes in neutrophils, including some that had not been reported to date (e.g., CCL4, TNFα, MAP3K8, IκBζ). However, only some of the corresponding proteins are similarly induced (e.g., CXCL8, CCL4). This involves several signaling pathways (e.g., Syk, TAK1, p38 MAPK, MEK/ERK, PI3K/Akt) and downstream effectors (transcription factors NF-κB, and possibly C/EBP and CREB as well). The same signaling pathways also participate in MSU-driven NET formation. Thus, our findings unveil several potentially important therapeutic targets for acute episodes of gouty arthritis, which feature a massive neutrophil influx. The fact that inhibitors for several of these molecular targets are already undergoing clinical trials (48–51) makes an eventual translation to the patient more than a remote possibility.

## Data Availability Statement

Datasets generated for this study are available upon request to the corresponding author.

## Ethics Statement

The studies involving human participants were reviewed and approved by Comité d'éthique de la recherche du CIUSSS de l'Estrie—CHUS Project #2001-18, 01-16. The patients/participants provided their written informed consent to participate in this study.

## Author Contributions

OT carried out the experiments for most aspects of the paper, compiled most the data, and wrote the first draft. TM compiled and analyzed the gene microarray data. VC completed experiments for Figures 5B, 6. MI and SS-P carried out the initial experiments for this project. CD provided conceptual input. PM designed the research, mentored the other authors, and wrote the final version of the paper.

### Conflict of Interest

The authors declare that the research was conducted in the absence of any commercial or financial relationships that could be construed as a potential conflict of interest.
