# Supplementary material for: Cytokine Production and NET Formation by Monosodium Urate-Activated Human Neutrophils Involves Early and Late Events, and Requires Upstream TAK1 and Syk
Source: Front Immunol. 2020 Jan 15;10:2996. doi: 10.3389/fimmu.2019.02996 (PMC6974451; doi:10.3389/fimmu.2019.02996)
Supplement: Supplementary file 1 [file Data_Sheet_1.PDF]

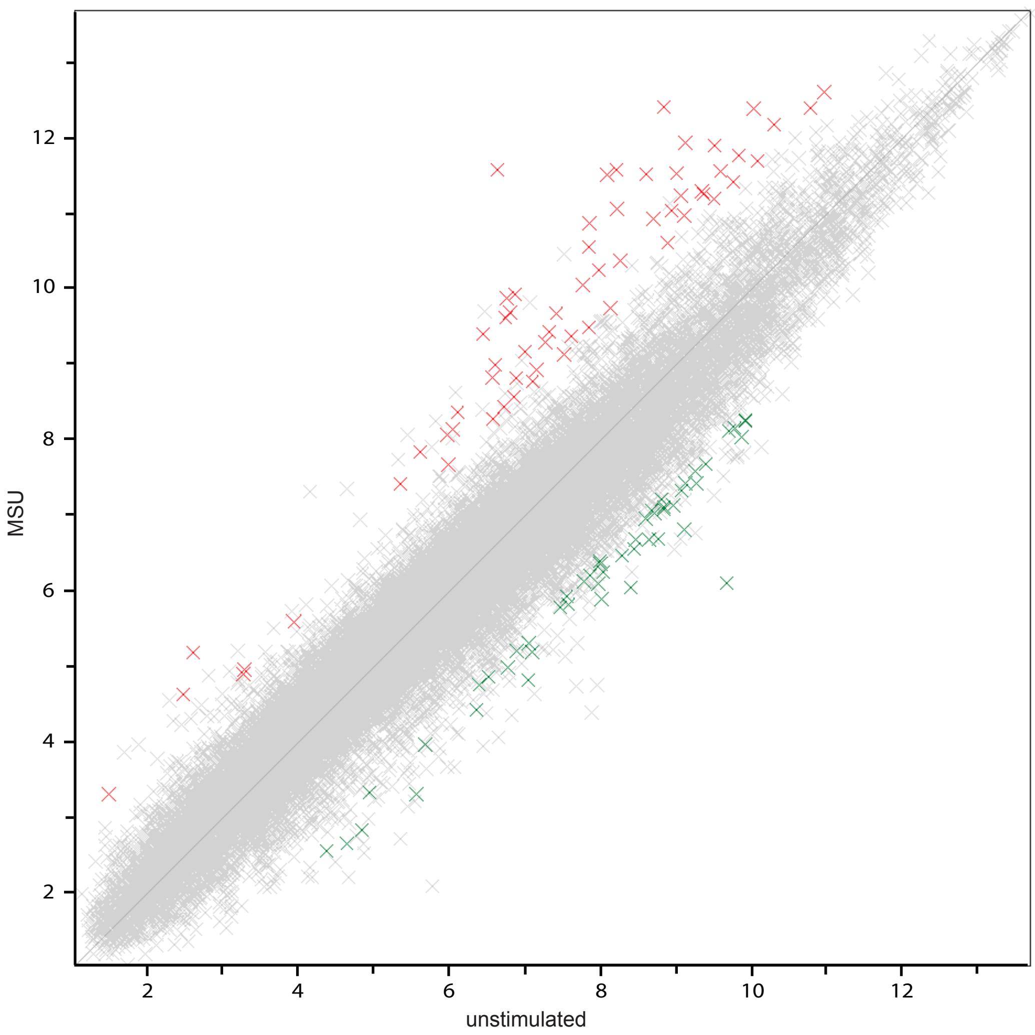

Supplementary Figure 1. Genomic changes elicited by MSU in human neutrophils.
Cells were stimulated with 1 mg/ml MSU (or diluent control) for 3 h at 37°C; total RNA was then extracted and 3 μg per experimental condition was pooled among 3 independent experiments and processed for gene microarray analysis. The scattergraph depicts the expression of various transcripts, relative to unstimulated controls. Colored crosses represent transcripts that are modulated 3-fold or more, for which the corresponding protein is known.

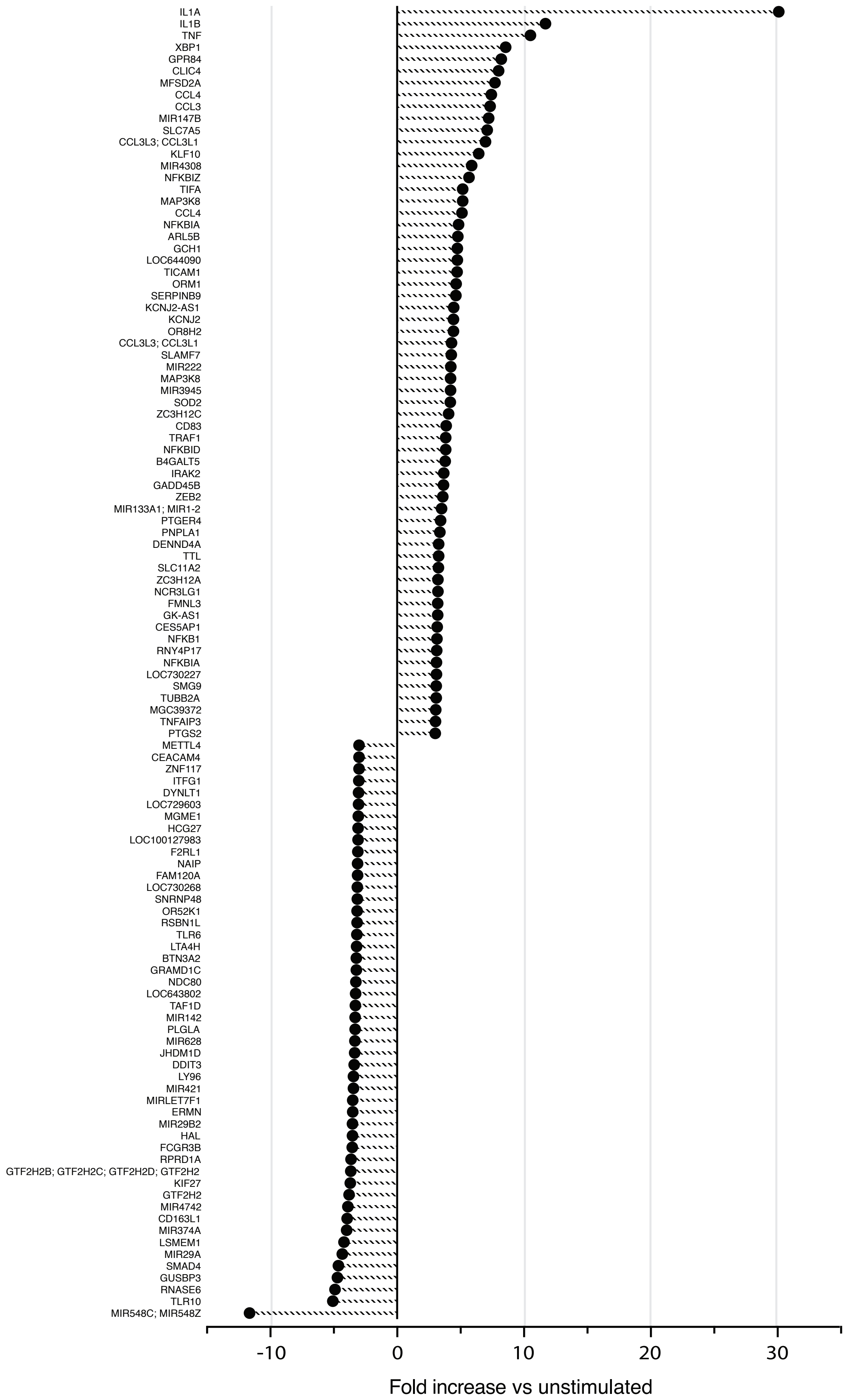

Supplementary Figure 2. Genomic changes elicited by MSU in human neutrophils.
Cells were stimulated and samples processed as decribed in Fig S1, and for genes with known corresponding proteins whose expression was modulated 3-fold or more, the extent of modulation is depicted.
